# Effectiveness of weekly cell phone counselling calls and daily text messages to improve breastfeeding indicators

**DOI:** 10.1186/s12887-018-1308-3

**Published:** 2018-10-30

**Authors:** Archana Patel, Priyanka Kuhite, Amrita Puranik, Samreen Sadaf Khan, Jitesh Borkar, Leena Dhande

**Affiliations:** 1 Department of Pediatrics, Indira Gandhi Government Medical College, Nagpur, Maharashtra 440018 India; 2 Lata Medical Research Foundation, Nagpur, Maharashtra 440022 India

**Keywords:** Breastfeeding counselling, Cell phone counselling, Exclusive breastfeeding, Infant nutrition, Lactation, Infant and young child feeding, Post-natal counselling, Maternal health

## Abstract

**Background:**

Every year, nearly one million deaths occur due to suboptimal breastfeeding. If universally practiced, exclusive breastfeeding alone prevents 11.6% of all under 5 deaths. Among strategies to improve exclusive breastfeeding rates, counselling by peers or health workers, has proven to be highly successful. With growing availability of cell phones in India, they are fast becoming a medium to spread information for promoting healthcare among pregnant women and their families. This study was conducted to assess effectiveness of cell phones for personalized lactation consultation to improve breastfeeding practices.

**Methods:**

This was a two arm, pilot study in four urban maternity hospitals, retrained in Baby Friendly Hospital Initiative. The enrolled mother-infant pairs resided in slums and received healthcare services at the study sites. The control received routine healthcare services, whereas, the intervention received weekly cell phone counselling and daily text messages, in addition to counselling the routine healthcare services.

**Results:**

1036 pregnant women were enrolled (518 - intervention and 518 - control). Rates of timely initiation of breastfeeding were significantly higher in intervention as compared to control (37% v/s 24%, *p* < 0.001). Pre-lacteal feeding rates were similar and low in both groups (intervention: 19%, control: 18%, *p* = 0.68). Rate of exclusive breastfeeding was similar between groups at 24 h after delivery, but significantly higher in the intervention at all subsequent visits (control vs. intervention: 24 h: 74% vs 74%, *p* = 1.0; 6 wk.: 81% vs 97%, 10 wk.: 78% vs 98%, 14 wk.: 71% vs 96%, 6 mo: 49% vs 97%, *p* < 0.001 for the last 4 visits). Adjusting for covariates, women in intervention were more likely to exclusively breastfeed than those in the control (AOR [95% CI]: 6.3 [4.9–8.0]).

**Conclusion:**

Using cell phones to provide pre and postnatal breastfeeding counselling to women can substantially augment optimal practices. High rates of exclusive breastfeeding at 6 months were achieved by sustained contact and support using cell phones. This intervention shows immense potential for scale up by incorporation in both, public and private health systems.

**Trial registration:**

This study was retrospectively registered with Clinical Trial Registry of India (http://www.ctri.nic.in/Clinicaltrials/pmaindet2.php?trialid=3060) Trial Number: CTRI/2011/06/001822 on date 20/06/2011.

## Background

Every year, suboptimal breastfeeding is responsible for around 800,000 under 5 child deaths globally [1]. It has been found to be the second largest risk factor for children under 5 years with 47.5 million disability-adjusted life years lost in the year 2010 [2]. Universal practice of exclusive breastfeeding has the potential to avert 11.6% of under -5 deaths [1]. All-cause neonatal mortality could be reduced by 22.3%, just by timely initiation of breastfeeding (defined by the World Health Organization as putting the newborn to the breast within 1 h of birth). Timely initiation of breastfeeding has the potential to save 250,000 newborns in India alone [3]. Infants who have delayed initiation of breastfeeding (initiation of breastfeeding > 1 h after birth) have 33% greater risk of neonatal mortality when compared to those with timely initiation of breastfeeding [4]. Exclusive breastfeeding protects against ear infections, allergies, anaemia in infants and has large ‘programming’ effects on risks for hypertension, hypercholesterolemia, obesity, cancer, autoimmune disease, and cognitive function later in life [5, 6].

Suboptimal feeding causes malnutrition which accounts for 10% of the global disability adjusted life years for under five children and 50% of the mortality [7]. Thus, in 2001, World Health Organization, after reviewing available evidence, made a global recommendation that all infants should be breastfed exclusively for 6 months and continue until 24 months. Breastfeeding should be supplemented with semi-solid or complementary foods after 6 months of age, as growth faltering may start with lack of timely initiation of complementary feeding [8–10]. Despite the known advantages of breastfeeding and timely initiation of complementary feeding, the Indian National Family Health Survey 2005–06 reported timely initiation of breastfeeding rates of 24.5%, exclusive breastfeeding rates at 6 months of 46.4%, and only 56.7% of 6–9 month old being fed complementary foods [11].

In India, there is an increase in the number of women delivering in hospitals due to a government monetary incentive scheme but the health staff has limited counselling skills for infant and young child feeding. Studies have shown that all infant and young child feeding indicators are better in women who adhere to their scheduled antenatal visits where they may have received breastfeeding related counselling during these visits [12]. A meta-analysis of individual peer counselling for the promotion of exclusive breastfeeding showed that the odds of exclusive breastfeeding in mothers receiving lactation counselling were substantially higher in the neonatal period (15 studies; odds ratio [OR] 3.45, 95% CI (2.20–5.42), *p* < 0.0001; random effects) and at 6 months of age (9 studies; 1.93, 95% CI (1.18–3.15), *p* < 0.0001) [13]. However, individualized counselling at health centres or by home visits, is expensive and not feasible in a populous, low income country, like India. Cultural barriers restrict women from leaving their households for at least 6 weeks after delivery and when required they need to be escorted by a care-giver to the facility.

In India, nearly all households including those below the poverty line have at least one cell phone. Given the extensive usage of cell phones, it is now possible to use it for health promotion and bringing about behavioural changes among the pregnant women and their families [14, 15]. Health workers can not only use cell phones to counsel pregnant women but also use the short text message system to send reminders and health promotional messages. Breastfeeding practices can also be enhanced through cell phone counselling. It can provide opportunity for early detection of breastfeeding problems, preventing of erroneous guidance by family members, friends, or health professionals, and, reduce the need to visit a hospital. We conducted a study to assess the effectiveness of using cell phones for personalized lactation counselling to improve exclusive breastfeeding rates. The aim of this study was to evaluate the effectiveness of text messages and counselling using cell phones as they are ubiquitous, even in the lower socio-economic strata of the urban population. Other forms of communications such as landlines, smart phones, internet, laptops etc. are not available in these poor households. The secondary objectives were to assess rates of timely initiation of breastfeeding, timely initiation of complementary feeding, pre-lacteal feeding, bottle feeding, infant hospitalization, satisfaction with the lactation counselling received and infant weight. We also evaluated the cost-effectiveness of cell phones to increase exclusive breastfeeding rates at 6 months of infant’s age.

## Methods

### Trial design, settings and location

This was a two arm, hospital-based pilot study conducted in four urban, public, maternity hospitals in Nagpur, India from August 2010 – to June 2012. This pilot study was conducted to understand the effectiveness of weekly cell phone counselling and daily text messages meant for pregnant and lactating women attending antenatal care and infant immunization clinics at these hospitals. This pilot will be essential to design a larger cluster randomized control trial to be implemented in rural India.

### Eligibility criteria

The participating hospitals (two in intervention and two in control) had to have annual deliveries of above 5000 and catered to women belonging to poor socio-economic background. Women in their third trimester (32–36 weeks), registered for antenatal clinics, planning to deliver at the same hospital and willing to give follow up till 6 months of infant age were considered eligible. An informed consent was obtained from all eligible women. Women with presence of complications in pregnancy that could affect exclusive breastfeeding such as severe anemia (Hb < 6 g/dL), at the risk of eclampsia or pre-eclampsia, consuming drugs contraindicated in pregnancy or HIV positivity were excluded. A record of all women screened, consented and attrition was maintained, including those ineligible and the reasons for not participating in the study.

### Randomization

Standardized Baby Friendly Hospital Initiative re-training was imparted by certified instructors to healthcare providers at all the four hospitals using the ‘Breastfeeding Promotion Network of India’ curriculum. The hospitals were then randomized to intervention (cell phone counselling + Baby Friendly Hospital Initiative re-training) and control (Baby Friendly Hospital Initiative re-training only) by the toss of a fair coin.

### Description of the intervention

Cell phone counselling was provided by certified lactation counsellors once a week, starting in the third trimester of pregnancy until a week after the infant was 6 months old. These counsellors were auxiliary nurse midwives with additional training for counselling over the phone. They provided advice on importance of antenatal care, iron-folic acid supplementation, maternal nutrition, appropriate infant and young child feeding practices, avoiding of pre-lacteal feeds (additional liquid supplements prior to initiation of breastfeeding), how to deal with problems regarding breastfeeding and infant immunizations. The counsellors also facilitated seeking of care at the hospitals if the mother or infant reported ill. Additionally, women received a text message daily, in the regional language to augment appropriate feeding practices. These women were also provided cell phones, seven free recharge vouchers and subsidized prepaid calling cards. Also, they could call the counsellors as and when needed, using a speed dial facility. During the study, if a mother lost her study cell phone, she was asked to use her personal or family cell phone.

The counsellors were trained to manage their counselling logs for scheduling their weekly calls and sending daily health promotional bulk text messages.

### Implementation and data collection

Prior to randomization, the baseline exclusive breastfeeding rates at the participating hospitals were assessed at delivery; 6, 10, 14 weeks postpartum. The rates of exclusive breastfeeding 24 h post delivery were 71.8% in the intervention and 72.3% in the control; similarly, at 6 weeks the rates were 52.8% versus 64.3%, at 10 weeks 52% versus 65% and at 14 weeks they were 40.3% versus 57.6% respectively. Data were collected by independent, trained data collectors from the enrolled women at the study hospitals. These data collection visits coincided with the woman’s antenatal care and child immunization visits. Data were collected at the following time points – registration (visit 1), a week after registration (visit 2), within 24 h of delivery (visit 3) and at 6 weeks (visit 4), 10 weeks (visit 5), 14 weeks (visit 6) post delivery of a live birth. The last two visits were at 6 months (visit 7) and a week after 6 months (visit 8). At registration information on socio-demographic details and preliminary health status were collected. At visit 2, information was collected on maternal illness, whether routine breastfeeding advice has been received and if breast examination has been done. In visit 3, data regarding mode of delivery, birth outcome, place of delivery, infant anthropometry, breastfeeding initiation, pre-lacteal feeds given along with their reasons, maternal or infant illnesses that prolonged hospitalization were obtained. In post-natal visits (4, 5, 6, 7 and 8) data were collected on breastfeeding practices, infant immunization and initiation of complementary feeding. Maternal satisfaction was noted, in both arms, by using a pictorial Likert scale. Random unannounced home visits in 5% of the intervention sample were conducted by data collectors to assess exclusive breastfeeding and inquire from family members about presence of infant formula or bottle in the household.

### Cost data collection

The health care costs incurred by the healthcare provider and patients were collected using micro–costing techniques. These costs were measured at enrollment, at delivery and on any subsequent hospitalization (maternal or infant). The costs of cell phones, caller plan subscription, text messages, dialed calls and recharge were recorded. The time and salaries of lactation counsellors, costs of health facility visits and hospitalizations were noted. The variable costs, i.e., direct medical (defined as cost of service, investigations and medication), direct non-medical (defined as cost of travel, food, living outstation etc.) and indirect costs (defined as wages lost during hospital visits) of the two study arms, were measured. The protocol driven costs were excluded from cost calculations. The mean differences in costs and the predictors of total cost were analyzed. The incremental cost-effectiveness of the two study arms was assessed as the incremental total cost of intervention per percentage increase in exclusive breastfeeding.

### Outcomes

The primary outcome was exclusive breastfeeding rates at delivery, and postnatal 6, 8, 10, 14 weeks, 6 months and a week after 6 months. It was assessed using the standard World Health Organization’s 24-h recall questionnaire. An infant receiving only breastmilk and no supplemental liquids or solid foods other than vitamins, minerals supplements, medicines or *Janamghuti* (herbal supplement) in last 24 h was considered to be exclusively breastfed. Other outcomes assessed were timely initiation of breastfeeding (breastfeeding the infant within an hour of birth), pre-lacteal feeds (additional liquid supplements prior to initiation of breastfeeding), neonatal outcomes, bottle feeding rates (use of bottle with nipple / teat), timely initiation of complementary foods (initiation of semi-solid foods after completion of 6 months of infant’s age), infant hospitalizations (any hospitalization more than 24 h related to an illness), infant weight (unclothed weight to the nearest 10 g), maternal satisfaction and incremental cost-effectiveness.

### Sample size

We anticipated a total of 1036 mothers-infant dyads (518 per group i.e. 259 per cluster) would participate in the trial, based on the cluster sample size calculation and analysis plan (PASS 2007 software) to achieve 80% power and 5% two-sided alpha to detect an absolute difference between the group proportions of 0.15 (46% exclusive breastfeeding in control group under null hypothesis and 61% under the alternative hypothesis). The test statistic used was the two-sided Z test (unpooled) and the intra cluster correlation coefficient was 0.008.

### Data analysis

All the analyses were performed in STATA version 11.2, STATACorp, 4905, Lakeway drive, College Stations, Texas, United States of America. These analyses were conducted at the mother-infant dyad level, for both intervention and control arms (unclustered analyses). The primary analyses compared the prevalence of exclusive breastfeeding in children at 6 months using Pearson’s chi-square tests and 95% confidence intervals for the group differences. We used generalized linear mixed models for non-continuous outcomes (logistic mixed models for binary outcomes - percentage of exclusive breastfeeding). Modelling analyses examined the primary outcome variable taking into account the repeated measurements within children (time) as random effect and all co factors as fixed effects. Variables that may have had impact on the outcome based on a review of the literature were selected as covariates and adjusted for in the models.

Cost analysis was done by calculating the mean costs of cell phone use, counselling, the facility visits and inpatient stay if any. A robust boot-strap method was used to obtain the incremental cost effectiveness ratio. A re-sampling to 100,000 observations was done. Group differences in mean cost of the study arms were assessed using Student’s t-test after normalizing the data. For the incremental cost-effectiveness, the numerator was the difference in the predicted total costs and the denominator was the difference in effects such as the number of not exclusively breastfeeding avoided i.e. number of inappropriate practices that were avoided by an incremental cost of using cell phones.

## Results

**Fig. 1:**
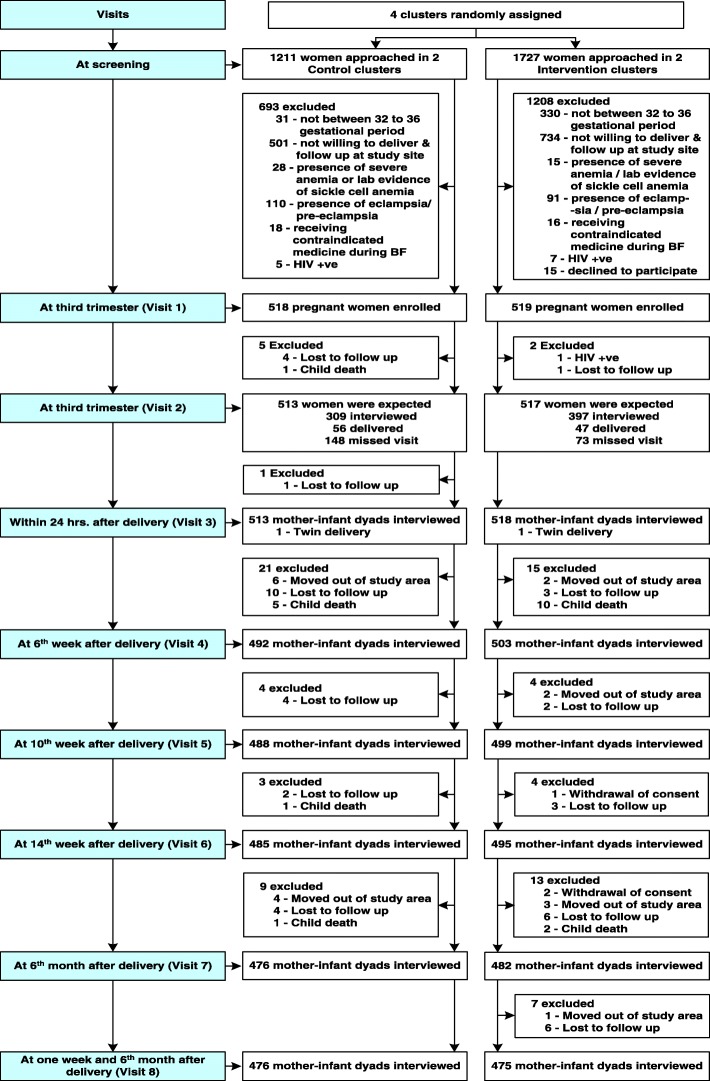
Flow chart of study recruitment and attrition

We screened 2938 pregnant women from the four hospitals and a total of 1037 were enrolled, of which 518 were assigned to the control group and 519 to the intervention group (Fig. 1).

After randomization of the study sites, baseline characteristics of women enrolled in the study were compared. Rates of low BMI (mother’s), Other Backward Classes (castes), age of mother (21–30 years), primigravida, decision making ability, advice received on breastfeeding at least once during antenatal period (by doctor or nurse), iron–folic acid supplementation, breast examination done by a doctor and advice from relatives were higher in control group as compared to the intervention. On the other hand, Muslim population, maternal education less than 10th grade, age of mother (< 21 years), infrequent exposure to mass media, ability to visit health facility alone, mean level of hemoglobin, mean number of antenatal visits and ownership of personal cell phones were higher in the intervention group (Table 1).

**Table 1:** Baseline characteristics of intervention and control arms post randomization

Maternal characteristics	Control (*N* = 518)		Intervention (*N* = 519)	
	N	%	n	%
Mothers age (y)	518		518	
<=24	285	55.1	264	50.9
25–30	201	38.7	213	41.0
> = 31	32	6.2	41	7.9
Mother’s BMI (kg/m^2^)	518		518	
< 18.5	48	9.3	26	5.0
> 18.5	470	90.8	492	95.0
Religion	518		519	
Hindu	367	70.9	331	63.8
Muslim	68	13.1	156	30.1
Christian	4	0.8	1	0.2
Sikh	1	0.2	0	0.0
Buddhist	77	14.8	31	6.0
Others	1	0.2	0	0
Caste	518		519	
Other backward classes(OBC)	199	38.3	206	39.7
Scheduled castes(SC)	106	20.4	67	12.9
Scheduled tribes(ST)	100	19.3	52	10.0
Others	114	22	194	37.4
Mother can read	518		519	
Yes	514	99.0	493	95.0
Mother can write	518		519	
Yes	513	98.8	499	96.2
Maternal education (y)	518		519	
< 10	86	16.6	127	24.5
10–12	277	53.4	239	46.1
> 12	156	30.1	153	29.5
Age at marriage (y)	518		517	
< 21	210	40.5	235	45.5
21–30	305	59.0	280	54.2
> 30	3	0.6	2	0.4
Working hours per day (Mean ± SD)	10	6.6 ± 3.5	22	4.4 ± 2.1
Household Wealth index	517		518	
Poorest	95	18.4	112	21.6
Poorer	116	22.4	94	18.2
Middle	136	26.3	127	24.5
Richer	85	16.4	75	14.5
Richest	85	16.4	110	21.2
Exposure to mass media	518		519	
Not at all/At least once a week	47	9.1	84	16.2
Almost everyday	471	90.9	435	83.8
Number of decisions participant make	518		519	
0	107	20.6	156	30.1
1–2	170	32.8	153	29.5
3–4	57	11.0	35	6.7
5–7	184	35.7	175	33.7
Allowed to visit market places	513		518	
Alone	12	2.3	35	6.8
With someone else/Not at all	501	97.7	483	93.2
Allowed to visit health facility	513		518	
Alone	10	2.0	36	7.0
With someone else/Not at all	503	98.1	482	93.1
Allowed to go outside the village / community	513		518	
Alone	6	1.2	16	3.1
With someone else/Not at all	507	98.9	502	96.9
Is she a primi gravida?	513		517	
Yes	287	56.0	256	49.5
Period of gestation (weeks) according to LMP (Mean ± SD)	510	33.8 ± 1.4	495	33.9 ± 1.3
Number of ANC visits attended (Mean ± SD)	516	6.1 ± 2.3	519	7.9 ± 3.6
She takes iron and folic acid supplementation	513		518	
Yes	461	89.9	432	83.4
She takes calcium supplementation	513		518	
Yes	428	83.4	431	83.2
Number of the tetanus immunization doses received (Mean ± SD)	514	2 ± 0.1	515	2 ± 0.2
Received advice on breastfeeding at least once during antenatal period	518		517	
Yes	125	24.1	51	9.9
Received the advice on breastfeeding from Doctor	518		517	
Yes	76	14.6	17	3.3
Received the advice on breastfeeding from Nurse	518		517	
Yes	36	6.9	4	0.8
Received the advice on breastfeeding from Social worker	518		517	
Yes	7	1.4	6	1.2
Received the advice on breastfeeding from Relative	518		517	
Yes	42	8.1	22	4.3
Breast examination done by Doctor /Nurse	518		517	
Yes	24	4.6	9	1.7
Breast problem on examination	518		516	
a) Flat nipple	1	0.2	1	0.2
b) Inverted Nipple	1	0.2	0	0.0
She breastfed her previous children	220		266	
Yes	180	81.8	218	82.0
Any obstetrics complication	518		515	
Yes	5	1.0	10	1.9
Any infections during pregnancy	518		515	
Yes	5	1.0	5	1.0
Any systemic illness^a^	517		515	
Yes	0	0.0	3	0.6
Hemoglobin level (g/dL)	518	9.9 ± 0.6	517	10.6 ± 0.9
Cell Phone Information				
Family have a phone	518		517	
Yes	503	96.9	505	97.7
Family have a Landline phone	513		515	
Yes	28	5.5	29	5.6
She is having cell phone for personal use	518		517	
Yes	215	41.4	243	47.0

^a^Any systemic illness was defined as any health related status or condition that was previously diagnosed by the physician and documented evidence for the same was present with the participant such as heart condition (congenital heart disease, rheumatic heart disease, Ischemic heart disease etc.), blood pressure, diabetes mellitus, UTI etc.)

### Exclusive breastfeeding

**Fig. 2:**
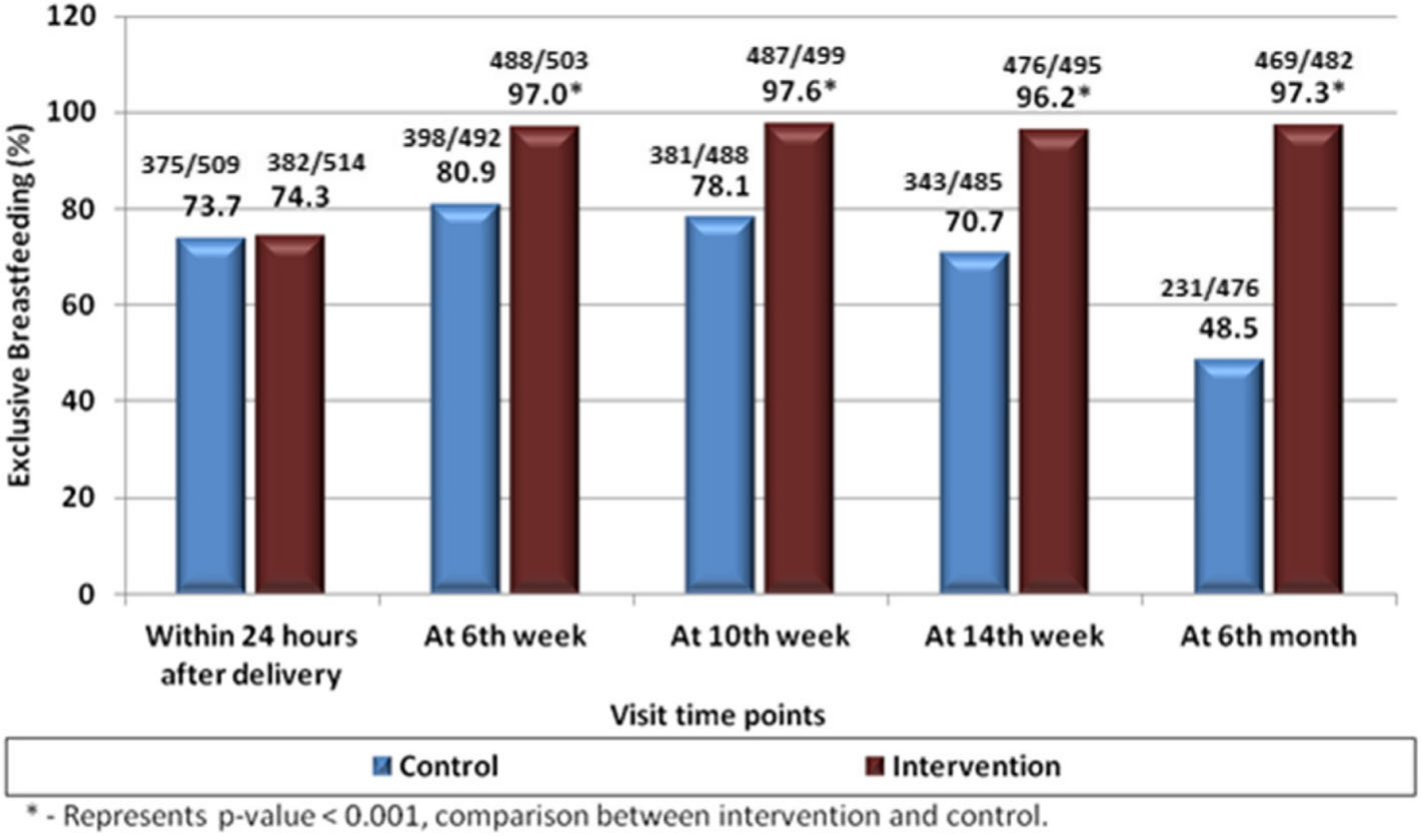
Rates of exclusive breastfeeding intervention v/s control

Comparable proportion of women in control and intervention were exclusively breastfeeding their infants within 24 h of delivery, with significant increase at subsequent visits in intervention. The rates of exclusive breastfeeding were sustained above 95% at all visits in the cell phone group but dropped from 81% at 6 weeks to 48.5% at 6 months in the control group. The distribution of women exclusively breastfeeding at each visit is shown in Fig. 2.

On multivariable analyses, significantly higher adjusted odds ratio was observed for exclusive breastfeeding was 6.30 (95% CI: 4.93, 8.03) in the cell phone intervention group when adjusted for the following covariates: mother’s age, BMI, religion, caste, education, age at marriage, household wealth index, exposure to mass media, household decision making power, parity, obstetric complications, possessing a personal cell phone, number of antenatal clinic visits, mode of delivery, place of delivery, sex of baby and low birth weight. Thus, each woman who received the intervention was six times more likely to exclusively breastfeed her infant for six months in comparison to those women who received standard healthcare services.

Overall, there were 506 out of 1031 women (49%) (control: 350/513; 68.2% and intervention: 156/518; 30.1%) that reported some reason for not exclusively breastfeeding at any given time point, starting from 24 h after delivery till 6 months of infant age. The intervention group had the highest rates of not exclusive breastfeeding on the first visit after delivery. The reasons were: woman’s choice to substitute breastmilk in 205/1031; 19.9% (control: 194/513; 37.8% vs. intervention: 11/518; 2.1%, *p* < 0.001), perceived insufficient breastmilk secretions in 129/1031; 12.5% (control: 88/513; 17.2% vs. intervention: 41/518; 7.9%, *p* < 0.001) and prescription of infant formula by physicians in 131/1031; 12.7% (control: 85/513; 16.6% vs. intervention: 46/518; 8.9%, *p* < 0.001). Infant illness was reported in 77/1031; 7.5% (control: 45/513; 8.8% vs. intervention: 32/518; 6.2%) of cases of mothers not exclusively breastfeeding, whereas, maternal illness was reported in only 8/1031; 0.8% (control: 5/513; 1.0% vs. intervention: 3/518; 0.6%) of cases.

### Timely initiation of breastfeeding, pre-lacteal feeds and neonatal outcomes

Rates of initiation of breastfeeding within an hour of birth were significantly higher in the intervention compared to the control (36.9% v/s 23.6% *p* < 0.001). Reasons reported by the women for delayed initiation of breastfeeding were: caesarean section (419/1031; 40.6%); delayed mother-baby contact due to late shifting of the baby with the mother (294/1031; 28.5%); infant illness (86/1031; 8.3%); infant had poor suck (30/1031; 2.9%); perceived insufficient breastmilk secretions (26/1031; 2.5%); breast related problems (6/1031; 0.6%); choice of the woman to substitute breastmilk (5/1031; 0.5%) and maternal illness (1/1031; 0.1%). The rates of pre-lacteal feeds were similar in both groups (intervention: 19%, control: 18%). A comparison of mother and newborn characteristics at birth between control and intervention is explained in Table 2.

**Table 2:** Comparison of maternal and newborn characteristics within 24 h of delivery

Child characteristics	Control (*N* = 513)		Intervention (N = 518)	
	N	%	N	%
Mode of Delivery	513		518	
Normal	280	54.6	311	60.0
Lower Segment Caesarean Section	230	44.8	202	39.0
Forceps/Assisted delivery	3	0.6	5	1.0
Place of Delivery	513		517	
Hospital	506	98.6	512	98.8
Home	5	1.0	5	1.0
Other	2	0.4	0	0.0
Status of Mother within 24 h	500		515	
Alive and well	499	99.8	514	99.8
Alive and sick	1	0.2	1	0.2
Maternal death	0	0.0	0	0.0
Present pregnancy outcome	513		518	
Single	511	99.6	516	99.6
Twin	2	0.4	2	0.4
Triplets or more	0	0.0	0	0.0
Birth Outcome	512		518	
Live birth	510	99.4	514	99.2
Fresh Still birth	1	0.2	2	0.4
Macerated SB	1	0.2	2	0.4
Sex of the baby	511		517	
Male	256	49.9	268	51.7
Status of baby within 24 h	511		516	
Alive and well	454	89.2	496	96.1
Alive and sick	53	10.4	16	3.1
Neonatal death	2	0.4	3	0.6
Low birth weight	503		517	
Yes	106	21.1	102	19.7
Gestational age	504	39.3 ± 1.3	493	39.4 ± 1.8
Baby cried immediately after birth	511		515	
Yes	476	93.1	503	97.7
Was resuscitation of new born required	505		513	
Not required	469	92.9	503	98.1
With bag & mask	25	5.0	3	0.6
Intubation required	2	0.4	2	0.4
Not Known	9	1.8	5	1.0

### Bottle feeding

**Fig. 3:**
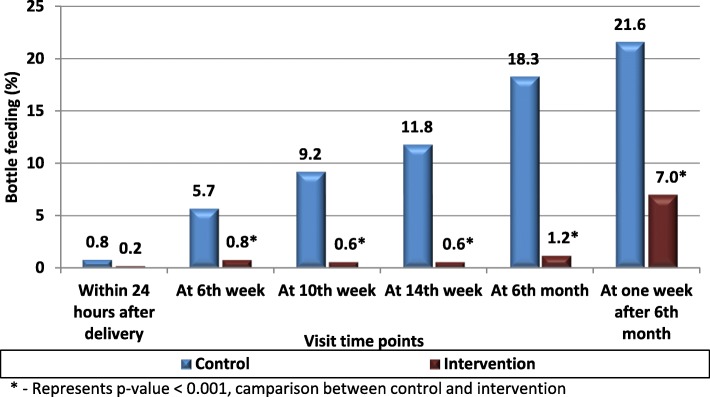
Rates of bottle feeding, intervention v/s control

The bottle feeding rates were negligible in the intervention group in the first 6 months whereas a steady increase, from 5.7% at 6 weeks to 18.3% at 6 months, was observed in the control cluster (Fig. 3).

### Timely introduction of complementary foods

Inappropriate introduction of complementary foods was observed in 26.9% in the control and only 0.4% in the intervention. In the intervention, nearly all the infants were introduced complementary foods appropriately (after completing 6 months) (99.6%).

### Infant hospitalization

The rates of infant hospitalization (neonatal intensive care unit admissions) were significantly lower in the intervention at visit 3 (12.5% v/s 6.8% *p* < 0.01). These rates were similar between both groups from visit 4 (6 weeks postnatal) till the last visit, with an exception of visit 7 where rates of hospitalization were greater in the intervention. (visit 4: 4.27% vs 5.77%, *p* = 0.28; visit 5: 1.02% vs 0.8%, *p* = 0.72; visit 6: 1.03% vs 1.62%, *p* = 0.42; visit 7: 1.26% vs 3.11%, *p* = 0.05; visit 8: 0.21% vs 0.84%, *p* = 0.18 in control vs intervention, respectively).

### Infant weight

The mean weight of babies at delivery was similar in both groups, but infants in the intervention group weighed significantly more than those in the control group at each subsequent visit (control vs intervention: visit 3, at birth: 2726 g vs 2730 g, *p* = 0.87; visit 4, at 6 weeks: 4085 g vs 4296 g, *p* < 0.001; visit 5, at 10 weeks: 4941 g vs 5204 g, *p* < 0.001; visit 6, at 14 weeks: 5710 g vs 5893 g, *p* < 0.001; visit 7, 6 months: 7183 g vs 7396 g, *p* = 0.026; visit 8, 6mo + 1 week: 7183 g vs 7396 g, *p* = 0.02).

### Maternal satisfaction with breastfeeding counselling

In the intervention, 92.3% of the women were completely satisfied with breastfeeding counselling provided by the lactation counsellors over cell phones. It was reported by 93% women from the intervention that the information received by them was helpful. In the control, only 36% of women were completely satisfied with the breastfeeding counselling provided by the health care provider and 31% felt that all the information they received regarding breastfeeding was helpful.

### Costs effectiveness

The average total cost incurred by all the subjects in the study from third trimester to 1 week and 6 months after delivery was Rs.4687.

The point estimate of incremental cost-effectiveness ratio showed that it was costlier [5603; 95%CI (5587, 5619)] to receive cell phone counselling (Table 3). The bootstrap estimate of the total mean cost of intervention group i.e. of cell phone counselling group [Rs. 6077; 95% CI (6074, 6080) versus Rs.3282; 95%CI (3279, 3284)] was more and the effect size i.e. proportion of exclusive breastfeeding at 6th month after delivery was significantly larger [0.95; 95%CI (0.95, 0.95) versus 0.42; 95%CI (0.42, 0.42)].

**Table 3:** Summary results of 100,000 bootstrap re-sampled observations of cost and effects

Variable	Mean	Std. Err.	[95% Conf. Interval]
Cost in Control group	3281.69	1.15	(3279.43, 3283.94)
Cost in Intervention group	6077.17	1.60	(6074.03, 6080.29)
Proportion of exclusive breastfeeding in Control	0.42	0.00	(0.42, 0.42)
Proportion of exclusive breastfeeding in Intervention	0.95	0.00	(0.95, 0.95)
ICER	5603.36	8.20	(5587.29, 5619.43)

## Discussion

This is the first trial using cell phones for breastfeeding counselling in India. We found that our cell phone intervention resulted in substantially higher rates of exclusive breastfeeding from the infant’s birth till 6 months of age. There were significant improvements in rates of initiation of breastfeeding as well as complementary feeding. Significant reductions in bottle feeding rates, from birth till a week after 6 months of age were also observed. Rates of pre-lacteal feeding were similar amongst both the groups. The intervention was also associated with lower rates of infant hospitalization within 24 h of delivery, increased maternal satisfaction and resulted in significantly better infant weight at all visits after birth.

The National Family Health Survey III (2005–2006) reported that 23.4% newborns had timely initiated breastfeeding (soon after birth) and only 50% of the infants (between 0 to 5 months) were exclusively breastfed [15]. Our study showed higher rates of exclusive breastfeeding at all the post-natal visits until 6 months in intervention and control arms. Exclusive breastfeeding rates in the intervention were remarkably high, over 95% in all the visits, as compared to the control in which only 48.5% of infants were exclusively breastfed. A recent systematic review concluded that, any pre and postnatal breastfeeding promotion strategy, increased exclusive breastfeeding rates by nearly 6 folds as compared to no intervention being provided [16]. Our intervention had a retention rate higher than that reported by a Cochrane review, where 50.9% of those receiving the intervention had stopped any breastfeeding by 6 months as opposed to 55.5% in the control (unweighted percentage) [17].

The remarkable improvement in exclusive breastfeeding rates observed during this study can be attributed to the frequency of support provided to the lactating mothers by daily text messages and weekly counselling calls made by the counsellors. The intervention may have also affected exclusive breastfeeding rates through other causal pathways. Prompt support received from the lactation counsellors, prevents the women from accepting incorrect advice given by their family members or friends. Also, during any illnesses, timely advice provided by the counsellors could limit further deterioration of their health which may otherwise impede breastfeeding. The frequent reinforcement of standard feeding recommendations by the lactation counsellors sustains, enables, and improves exclusive breastfeeding.

The control group too showed rates of exclusive breastfeeding higher than the national estimates. This increase can be attributed to the baby friendly hospital initiative retraining conducted for health providers at both control and intervention hospitals. Reasons observed for not adhering to exclusive breastfeeding (in both arms) were woman’s choice to supplement breast milk with other feeds; perceived insufficiency of breastmilk secretions and lastly prescription of infant formula by doctors. These reasons are amenable to frequent counselling and could be adequately addressed by the lactation counsellors resulting in excellent rates of exclusive breastfeeding in the intervention. Despite presence of the Infant Milk Substitute Act, rates of prescribing infant formula by a health practitioner continued to be higher in the control as compared to the intervention. This change may be attributed to the increased awareness of the women towards harmful effects of these supplements, as a result of frequent counselling.

High rates of exclusive breastfeeding noted in our study could also be a result of the Hawthorne effect. An attempt was made to mitigate this by conducting unannounced home visits in a sub-sample of mothers of the intervention arm. However, none of the home visits revealed that women practiced a behavior contrary to what they reported. The Hawthorne effect cannot be completely eliminated as these home visits were conducted in a sub sample.

The rates of timely initiation of breastfeeding were significantly higher in the intervention at 37% as compared to 24% in the control. Despite pre-natal counselling, the rates were much below the desired target of Millennium Development Goal of 50% [18]. These rates are dependent on behavior of hospital staff and less reliant on what the mother may desire as a result of her counselling. Therefore, prenatal cell phone counselling failed to have a desired impact. A major reason for the low rates of initiation was delayed shifting of the baby to the mother (28.5%) causing lower rates of skin to skin contact and noncompliance of essential newborn care recommendations [19]. This delayed shifting predominantly occurred in women who were delivered by caesarean section (40.6%), a known deterrent to timely initiation of breastfeeding [10, 20].

Cell phone counselling did not have an impact on reducing pre-lacteal feeds as changing family traditional practices over a short period of counselling, mostly directed at the mother, may not be sufficient [21].

The bottle-feeding rates were higher in the control with women starting to bottle feed their babies as soon as 6 weeks after birth. This occurrence was consistent with reports from other studies that have shown women with inadequate postnatal breastfeeding support, have a decline in exclusive breastfeeding rates and are at an increased risk of bottle feeding at about 6 weeks [22]. Face to face or cell phone counselling has shown to reduce bottle feeding rates and effectively increase duration of breastfeeding [16]. This study also showed that mothers appropriately started semi-solid foods after 6 months as a result of weekly cell phone counselling and daily text messages. Mothers from the control arm had inappropriately initiated complementary foods i.e. before 6 months further lowering exclusive breastfeeding rates to 46%. Similar studies have reported that sustained encouragement, confidence building and reassurance of mothers regarding the adequacy of their milk, both in terms of nutrition and quantity, has restricted the use of any other form of feeding beside breastfeeding till 6 months of infant age [23]. A women’s confidence in the adequacy of their feeds tends to erode in absence of sustained counselling and support, resulting in additional feeding earlier than 6 months due to family and peer pressure [24].

Over 93% of the women were satisfied with the weekly cell phone counselling they received, translating into high adherence to exclusive breastfeeding. Nutritional sufficiency of exclusive breastfeeding along with appropriate initiation of complementary feeding were also evident as weight gain of infants in the intervention was significantly better as compared to control. Similar results were reported by Thakur in Bangladesh in low birth weight babies [25, 26].

Lower rates of hospitalization into the neonatal intensive care unit (NICU) within the first 24 h of delivery in the intervention group may have resulted due to timely telephonic consultations received just around delivery. Women perhaps reported promptly to the hospitals, which may have resulted in better intra-partum care and fewer rates of neonatal resuscitation. This shows that cell phone counselling had a favorable impact on the health care seeking behavior at the time of delivery. Subsequently, the rates of infant hospitalization were similar in both groups.

The cost effectiveness analysis showed that, for a cost of Rs.5603 (approximately, 127 dollars) a 50% improvement in exclusive breastfeeding (at 6 months) can be achieved. The cost in terms of ‘years of life saved’, as a result of improvement of exclusive breastfeeding, estimated using the Markov model, was not within the scope of this study. This intervention, though being marginally costlier, has twice the potential to improve exclusive breastfeeding as compared to the existing healthcare services. It was found cost effective when compared with the World Health Organization’s CHOICE (CHOosing interventions that are Cost Effective) thresholds for low income countries [27]. For India the cost effectiveness threshold is equivalent to 1345 dollars that is the gross domestic product, per capita in year 2010 [28]. In comparison to this, our intervention costs below 130 dollars per mother–infant dyad.

This is one of the first trials where cell phones were used for lactation counselling to improve optimal feeding practices soon after birth. The impact of intervention on exclusive breastfeeding was adjusted for the differences in baseline characteristics. Therefore, the large and significantly beneficial impact of cell phone counselling, on breastfeeding indicators was not likely by chance. This was a pragmatic effectiveness trial that leveraged on the services of existing hospital staff therefore has potential to scale up in low resource setting. The cost-effectiveness assessment helped to inform investments needed for promotion of infant and young child feeding at these health facilities.

The limitation of the study was that it was an unblinded pilot study of only four clusters. This can potentially bias the results due to imbalances in baseline characteristics that may impact exclusive breastfeeding. However, the intervention arm had lower baseline rates of exclusive breastfeeding and other characteristics that may positively influence rates of exclusive breastfeeding. We adjusted for these imbalances by using generalized linear mixed model. We acknowledge that there were other unobserved differences between the hospitals and so residual confounding may remain.

Another limiting factor was that the intervention was not designed to assess the effectiveness of different frequencies of contact with the women, on exclusive breastfeeding. Ascertaining optimal frequency of contact needed to observe a similar improvement in exclusive breastfeeding rates was beyond the scope of this study. However, it has been noted that, if a woman is not provided prompt assistance, within few days, she is likely to adopt alternative inappropriate feeding practices. Other implementation challenges such as switched off phones, discharged phones, rejected calls or unanswered calls, calls received by someone other than the enrolled women and loss of cell phones were also encountered during the study.

## Conclusions

In conclusion, we found lactation counselling using cell phones proved to be a very useful tool for frequent and sustained support to pregnant and lactating mothers. It alleviates the need for hospital visits and face to face counselling. This intervention can be successfully implemented in low resource settings by training nurse midwives who can potentially communicate with large number of beneficiaries. This health care delivery system is now particularly relevant as the use of cell phones in Indian households is nearly universal. It needs further evaluation prior to scale up and incorporation into the public as well as private health systems.

## Abbreviations

ANC: Antenatal Care
BMI: Body Mass Index
DALYs: Disability Adjusted Life Years
Hb: Hemoglobin
HIV: Human Immunodeficiency Virus
ICER: Incremental cost effectiveness ratio
